# Protein Kinase D2 Regulates GRASP65 Phosphorylation and Golgi Ribbon Unlinking During G2/M Transition

**DOI:** 10.3390/cells15060565

**Published:** 2026-03-21

**Authors:** Inmaculada Ayala, Daniela Spano, Antonino Colanzi

**Affiliations:** Institute of Endotypes in Oncology, Metabolism and Immunology “G. Salvatore” (IEOMI), National Research Council (CNR), Via P. Castellino 111, 80131 Naples, Italy

**Keywords:** Golgi complex, PKD, GRASP65, cell cycle

## Abstract

**Highlights:**

**What are the main findings?**

- PKD2 is an upstream regulator of JNK2-dependent GRASP65 phosphorylation at serine 274 during the G2 phase.
- PKD2 activity is required for Golgi ribbon unlinking and proper G2/M progression.

**What are the implications of the main findings?**

- A PKD2–JNK2–GRASP65 signaling axis links Golgi remodeling to mitotic entry.
- The newly developed phospho-specific GRASP65 antibody enables investigation of upstream Golgi regulatory pathways in cell cycle control and disease.

**Abstract:**

The Golgi complex undergoes dynamic remodeling during the cell cycle, as ribbon unlinking in G2 is required for proper mitotic progression. Failure to fragment the ribbon leads to G2 arrest, whereas forced mitotic entry with intact ribbons results in multipolar spindle formation. Phosphorylation of the Golgi matrix protein GRASP65 at serine 277 (S277) in rat (S274 in human) by JNK2 is essential for ribbon unlinking, but its upstream regulation has remained unclear. Here, we generated and validated a phospho-specific antibody recognizing human GRASP65 phosphorylated at S274, enabling accurate detection of this modification. Using this tool, we identify protein kinase D2 (PKD2) as a critical upstream regulator required for GRASP65 phosphorylation and Golgi unlinking. GRASP65-S274 phosphorylation increases during G2 and is markedly reduced upon PKD2 inhibition or depletion, resulting in decreased Golgi unlinking and delayed G2/M transition. Conversely, PKD2-activating stimuli, including phorbol esters and nocodazole, enhance GRASP65 phosphorylation in a PKD2-dependent manner. These findings define PKD2 as a key regulator of the JNK2–GRASP65 signaling axis controlling Golgi disassembly at the G2/M transition. Moreover, the phospho-specific GRASP65 antibody described here provides a valuable tool to dissect upstream signaling mechanisms and to identify the initial triggers driving Golgi unlinking at G2 entry.

## 1. Introduction

The Golgi complex (GC) serves as a central hub for lipid synthesis, post-translational modifications of proteins and lipids, and the sorting and delivery of cargo to their final destinations [1]. Beyond its classical roles, the GC is increasingly recognized as a platform for integrating diverse signaling pathways [2,3]. In most vertebrate cells, the GC is organized as polarized stacks of flattened cisternae (from *cis* to *trans*) connected by tubular bridges (non-compact zones), forming a continuous network termed “Golgi ribbon”, typically positioned around the microtubule-organizing center (MTOC) [4,5].

Although the GC function is evolutionarily conserved, its structural organization varies among species. In *Saccharomyces cerevisiae*, the GC consists of isolated cisternae dispersed in the cytoplasm [6]. In contrast, in *Toxoplasma gondii*, it appears as a single stack [7]. In plants and *Drosophila*, multiple stacks are scattered throughout the cytoplasm [8,9]. In mammals, exceptions to the ribbon structure exist in differentiated muscle cells [10], oocytes [11], gastric parietal cells [12], uroepithelial cells [13], and neurons, in which the GC also includes isolated stacks as dendritic outposts [14].

The GC undergoes rapid remodeling in response to physiological cues [15,16]. During the cell cycle, the Golgi ribbon is progressively disassembled. In G2, it is unlinked into separate stacks [17]. During mitosis, these stacks fragment into vesicular-tubular clusters and ultimately into the “Golgi haze” in metaphase [17]. Reassembly begins during telophase, leading to the reformation of Golgi ribbons into the two daughter cells after cytokinesis [18]. Pre-mitotic unlinking is essential because its inhibition causes G2 arrest and mitotic failure [19], underscoring the concept of a “Golgi checkpoint” that ensures proper spindle formation and GC segregation [20]. Notably, forced mitotic entry with an intact ribbon results in defects such as spindle multipolarity and binucleation [21], which can potentially lead to genomic instability and tumorigenesis [22,23].

The structural integrity of the GC relies on a detergent- and salt-resistant scaffold called the “Golgi matrix” [24], composed mainly of golgins and GRASP proteins [25,26]. Golgins are anchored to the GC via their C-terminal domains, and they also tether membranes through their extended N-terminal coiled-coil regions [27]. GRASP65 and GRASP55 are matrix proteins recruited to the *cis*- and medial/*trans*-Golgi via complexes with GM130 and Golgin-45, respectively [28]. The GRASPs contain two conserved N-terminal PDZ domains involved in homodimerization and a C-terminal serine/proline-rich (SPR) region implicated in regulation processes [26,28]. These proteins tether cisternae into stacks and support the formation of the Golgi ribbon, the assembly of which is mediated by the formation of membrane tubules extending from the cisternal rims that dock with and fuse to homologous cisternae of adjacent stacks [29,30]. The docking step is mediated by GRASPs, likely through their ability to *trans*-oligomerize. Phosphorylation of specific sites in GRASP55 and GRASP65 by ERK and JNK2, respectively, inhibits the formation of these *trans*-oligomers, leading to the ribbon unlinking. Notably, inhibition of GRASP55 or GRASP65 phosphorylation prevents progression through the G2/M transition [17,31,32].

Significantly, members of the protein kinase D (PKD) family have been shown to promote Golgi unlinking by acting upstream of MEK1 and ERK [33]. The PKD family comprises the Ca^2+^/calmodulin-dependent serine/threonine kinases PKD1, PKD2, and PKD3. These kinases share a modular structure with a regulatory N-terminal region and a catalytic C-terminal kinase domain. The regulatory region contains two tandem diacylglycerol (DAG)-binding cysteine-rich domains (C1a and C1b) and a pleckstrin homology (PH) domain with an autoinhibitory function. Under resting conditions, PKDs are distributed diffusely in the cytosol and, to a lesser extent, in the nucleus, GC, and mitochondria [34]. Canonical activation occurs via PKC-dependent phosphorylation of two serine (S) residues within the activation loop (S706/710 in PKD2), followed by autophosphorylation of a C-terminal residue (S876 in PKD2), which serves as a widely used readout of PKD activity. PKD2 also localizes to centrosomes during G2, where it stabilizes Aurora A and facilitates centrosome separation; its inhibition results in proteasome-dependent degradation of Aurora A and G2/M arrest [35,36].

Understanding how the signaling pathways regulate the phosphorylation of GRASP55 and GRASP65 is critical to elucidating the mechanisms that control Golgi ribbon unlinking during the G2 phase of the cell cycle [17,28]. Given that ERK directly phosphorylates GRASP55 [37], it is possible that PKD functions upstream to regulate GRASP55 phosphorylation during cell cycle progression [33,37]. In contrast, we previously demonstrated that during G2, rat GRASP65 is phosphorylated by JNK2 at S277 [38], a residue that corresponds to S274 in humans [39]. GRASP65 is phosphorylated at multiple residues during G2 and mitosis; however, most of these sites are phosphorylated at later mitotic stages by CDK1 or PLK1, and each phosphorylation contributes to distinct steps of Golgi disassembly [39]. Among these sites, Ser274 stands out as the most relevant readout for G2-specific Golgi unlinking, as it is the earliest mitotically phosphorylated residue, appearing already in late G2 [39]. Importantly, S274 phosphorylation inhibits the tethering activity [38] and destabilizes a subset of Golgi-associated microtubules, in particular acetylated microtubules, thereby promoting Golgi unlinking [40]. However, the upstream regulators of the JNK2-GRASP65 pathway remain unknown.

To address this, we investigated whether PKD acts upstream of GRASP65 phosphorylation. In this study, using a novel phospho-specific antibody raised against a human GRASP65 peptide containing phosphorylated S274, we demonstrate that PKD2 activity is essential for GRASP65 phosphorylation, Golgi unlinking, and timely G2/M transition. Taken together, our findings reveal that PKD2 is a key regulator of GRASP65.

## 2. Materials and Methods

### 2.1. Cell Culture and Synchronization

HeLa cells were from the European Collection of Authenticated Cell Cultures (ECACC) and were cultured in DMEM (Invitrogen, Carlsbad, CA, USA), supplemented with 10% fetal bovine serum (Biochrom, Cambridge, UK). The medium contained 2 mM L-glutamine, 1 U/mL penicillin, and 50 µM streptomycin (Invitrogen). Cells were grown in a controlled atmosphere with 5% CO_2_ at 37 °C.

### 2.2. Antibodies and Reagents

Thymidine, fibronectin from human plasma, SP600125, CRT0066101, kb NB142-70, PMA, UO126, PD98059 and Mowiol 4-88 were from Sigma-Aldrich (St. Louis, MO, USA). Recombinant human EGF was from Preprotech (Cranbury, NJ, USA). DMSO was obtained from Carlo Erba, and RO-3306 was from Calbiochem (San Diego, CA, USA).

The antibodies were obtained from the following sources: rabbit anti-phospho-GRASP65, which recognizes the human sequence DPLPGPGSPSHSAP (267/280), where S274 is phosphorylated, was produced by outsourcing (Covalab, Bron, France) (1:30). Polyclonal anti-GRASP65 (#PA3-910 1:1000) was from Thermo Fisher Scientific (Waltham, MA, USA). Mouse anti-GM130 antibody was purchased from BD Biosciences (San Jose, CA, USA, #610823, 1:1000). Mouse anti-GAPDH (#97166S, 1:2000), rabbit anti-phospho-SAP/JNK (81E11) (#4688 1:1000), JNK2 (#4672S, 1:1000), and PKD2 (D1A7) (#8188S, 1:1000) were from Cell Signalling Technology (Danvers, MA, USA). Rabbit anti-phospho-histone H3 was from Millipore (Burlington, MA, USA, #06-570, 1:300). Rabbit anti-pericentrin was from Abcam (Cambridge, UK, #ab4448, 1:300). Alexa Fluor 488-, 568- and 647-conjugated secondary antibodies, as well as Hoechst 33342, were purchased from Invitrogen.

### 2.3. Synchronization

For synchronization at the G2/M transition, the cells were treated using the double thymidine protocol. Alternatively, to block the cells at the G2/M border, 9 µM of the CDK inhibitor RO-3306 was added for a total of 20 h, as previously reported [41].

### 2.4. Immunofluorescence Microscopy

Cells were grown on fibronectin-coated glass coverslips at a concentration of 10 µg/mL. For labeling with the P-GRASP65 antibody, the cells were fixed on ice with cold 4% paraformaldehyde for 15 min, treated with 0.1% TX100 for 3 min, blocked with 0.5% bovine serum albumin, 0.1% saponin, and 50 mM NH_4_Cl in PBS for 20 min, and finally with 5% horse serum in PBS (Gibco, BRL, Waltham, MA, USA) for 1 h. Finally, the antibodies were incubated in blocking buffer overnight at 4 °C. Following the secondary antibodies at room temperature, the coverslips were mounted on glass microscope slides with Mowiol Sigma-Aldrich (St. Louis, MO, USA). Immunofluorescence samples were examined using a confocal laser microscope (Zeiss LSM980 confocal microscope system; Carl Zeiss, Gottingen, Germany) equipped with ×63 1,4 NA oil objective. Optical confocal sections were taken at 1 Airy unit, with a resolution of 2048 × 2048 pixels, exported as .czi files, and transformed into .TIF with Fiji. The images were cropped with Adobe Photoshop 25.11.0 and composed using Adobe Illustrator 28.6.

### 2.5. siRNA, Plasmids and Transfection

PKD2 was targeted using the duplex 5′-GGCAGUUUGGAGUGGUCUA[dT][dT]-3′ (kindly provided by Cell Signalling Technology). The non-targeting sequence was 5′-UUCUCCGAACGUGUCACGU[dT][dT]-3′ [40]. The siRNA Silencer Select targeting the transcript variant of PKD2 was obtained from Ambion, Austin, TX, USA (now part of Life Technologies, Carlsbad, CA, USA, #s24646). The pcDNA3-YFP-PKD2 vector, expressing the human PKD2 fused to YFP, was purchased from GenScript (Nanjing, China, ID: 31821). The pcDNA3-YFP-PKD2 siRNA-resistant construct was generated by site-directed mutagenesis. The primers used for the mutagenesis reaction were as follows: 5′-CCTGACGAAGTGCTGGGCTCAGGTCAATTCGGCGTAGTATATGGAGGAAAACACCGGAAGACAGG-3′ and 5′-CCTGTCTTCCGGTGTTTTCCTCCATATACTACGCCGAATTGACCTGAGCCCAGCACTTCGTCAGG-3′. The GRASP65-EGFP-pcDNA3.1 vector, expressing human GRASP65 fused to EGFP, was generated by Restriction Free (RF) cloning as previously described (PMID: 20600952). Briefly, the EGFP coding sequence was amplified by PCR using two primers, each containing a target-specific sequence and a 5′ extension, overlapping the desired insertion sites at the destination vector. Subsequently, the double-stranded PCR product was used as a set of mega-primers for the second amplification reaction that allowed the cloning of the EGFP coding sequence downstream of the human GRASP65 coding sequence. The primers used for the amplification of the EGFP coding sequence were as follows: 5′-GACAGCCAGGCCCAGATCTCTACCACAGAATGTGAAGGCCCCTGGCCCGGGAT-3′ and 5′-AGGCACAGTCGAGGCTGATCAGCGGGTTTATTACTTGTACAGCTCGTCCATGCC-3′. The destination vector used in the RF cloning was GRASP65 pcDNA3.1(+)-C-Myc purchased from GenScript (ID: SC1200). The GRASP65 S274A-EGFP-pcDNA3.1 vector, expressing the human GRASP65-EGFP fusion protein in which the serine 274 in human GRASP65 was mutated to alanine, was generated by a site-directed mutagenesis reaction. The primers used for the mutagenesis reaction were as follows: 5′-CTTCCTGGGCCTGGGGCTCCCAGCCACAGTGC-3′ and 5′-GCACTGTGGCTGGGAGCCCCAGGCCCAGGAAG-3′. The PCR conditions for the RF cloning and the mutagenesis reactions were as follows: a single denaturation step of 5 min at 95 °C; 20 cycles of denaturation (95 °C, 1 min), annealing (57 °C for RF cloning; 60 °C for mutagenesis reactions, 1 min) and elongation (68 °C, 20 min); and a final elongation step of 10 min at 72 °C. The PCR reaction was carried out in a 50 µL final volume including the following components: 50 ng of DNA template, 200 µM of each dNTP, 1x PfuUltra High-Fidelity DNA Polymerase buffer, 0.2 µM of each primer for mutagenesis reactions or 150 ng of mega-primers for RF cloning, and 2.5 U of PfuUltra High-Fidelity DNA Polymerase (Agilent, Santa Clara, CA, USA). All constructs were verified by sequencing analysis. The siRNAs were transfected using Lipofectamine RNAiMAX (Invitrogen), according to the manufacturer’s instructions. Transfection of the pcDNA3-YFP-PKD2 siRNA-resistant construct, GRASP65-EGFP-pcDNA3.1, and GRASP65 S274A-EGFP-pcDNA3.1 plasmids was performed using Lipofectamine 2000 (Invitrogen), 24 h before fixation.

### 2.6. Western Blotting

Briefly, HeLa cells were lysed in ice-cold lysis buffer (50 mM Tris-HCl, pH 8, 150 mM NaCl, 1% TX-100, 0.1% SDS, 10% glycerol, 1 mM EDTA, 1 mM EGTA, 10 mM NaF, 20 mM β-glycerol phosphate supplemented with protease and phosphatase inhibitors) for 30 min. Subsequently, samples were collected and centrifuged at 14,000 rpm for 15 min at 4 °C. The supernatants were analyzed by SDS-PAGE. Chemiluminescent signals were detected using the ChemiDoc™ Imaging System (Bio-Rad, Hercules, CA, USA).

### 2.7. Quantitative Analysis

The fluorescence intensity of P-GRASP65 and GM130 was measured using Fiji software (version 2.16.0/1.54p) to normalize the data. Regions labeled with GM130 were outlined using a selection tool, and the “measure” function was used to quantify the area and integrated density of the fluorescence of P-GRASP65. The backgrounds of the images were corrected. Golgi objects and average size were measured employing Fiji by splitting channels, transforming into 8 bits, adjusting the threshold, converting them to binary, and selecting 50 random cells from 15 broad-field images. The results were obtained with the “analyze particles” function.

### 2.8. Statistical Analysis

Statistical analyses were performed using the Welch’s *t*-test to account for unequal variances between groups using the GraphPad Prism version 10.5.0.

## 3. Results

### 3.1. Characterization of an Antibody Against Phospho-GRASP65

We previously demonstrated that JNK2 phosphorylates S277 of rat GRASP65 during the G2 phase, thereby inducing Golgi unlinking [38]. In addition, we showed that inhibition of JNK by SP600125 or depletion of JNK2 leads to a strong and sustained G2 phase cell cycle arrest [38]. To investigate the upstream regulators of GRASP65 phosphorylation in human cells, we have generated a custom-made antibody against the peptide DPLPGPGSPSHSAP (aa 267-280), corresponding to a stretch within the serine/proline-rich (SPR) domain of the human sequence of GRASP65, which is phosphorylated at S274 [39].

**Figure 1:**
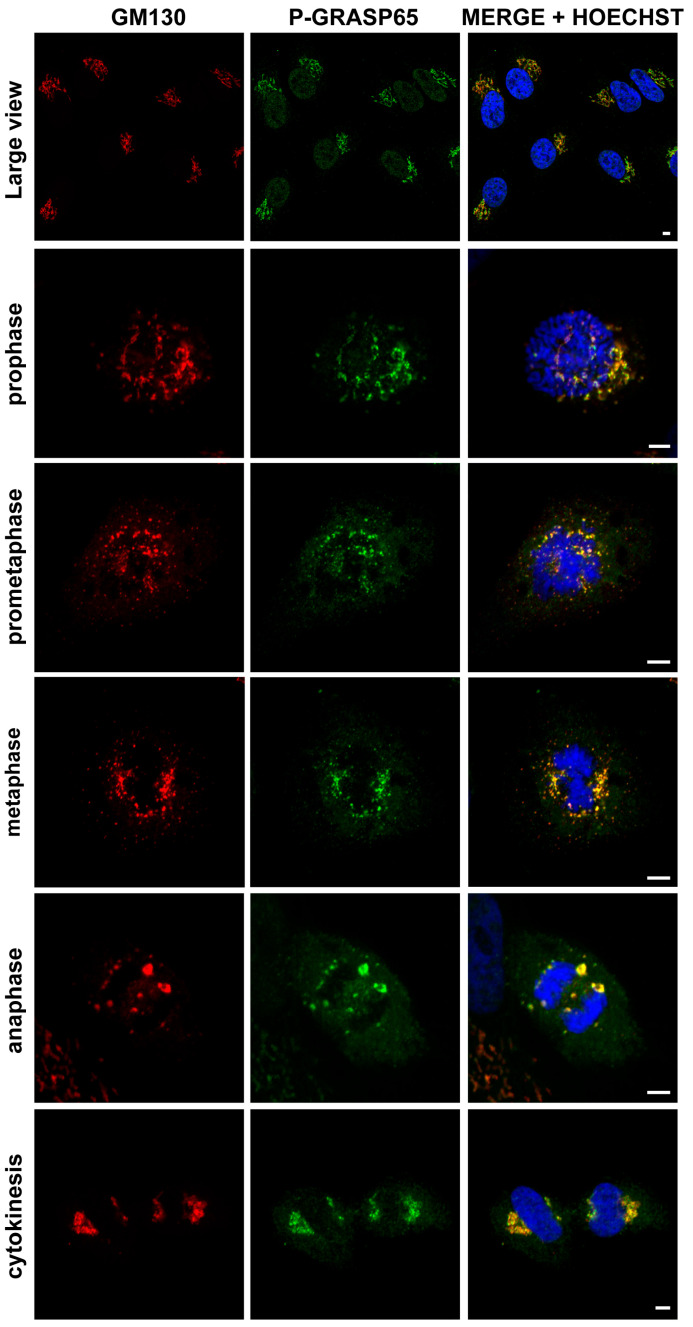
The P-GRASP65 antibody specifically labels the Golgi complex in HeLa cells. HeLa cells were synchronized using the double thymidine block and fixed at the mitotic peak. Cells were labeled with antibodies against P-GRASP65 (green) and GM130 (red) as a marker for Golgi localization. Hoechst 33342 (blue) was used to stain the DNA. The P-GRASP65 signal colocalizes with GM130. Representative images of different phases of the cell cycle are shown, as indicated in the figure. Scale bars: 5 µm.

**Figure 2:**
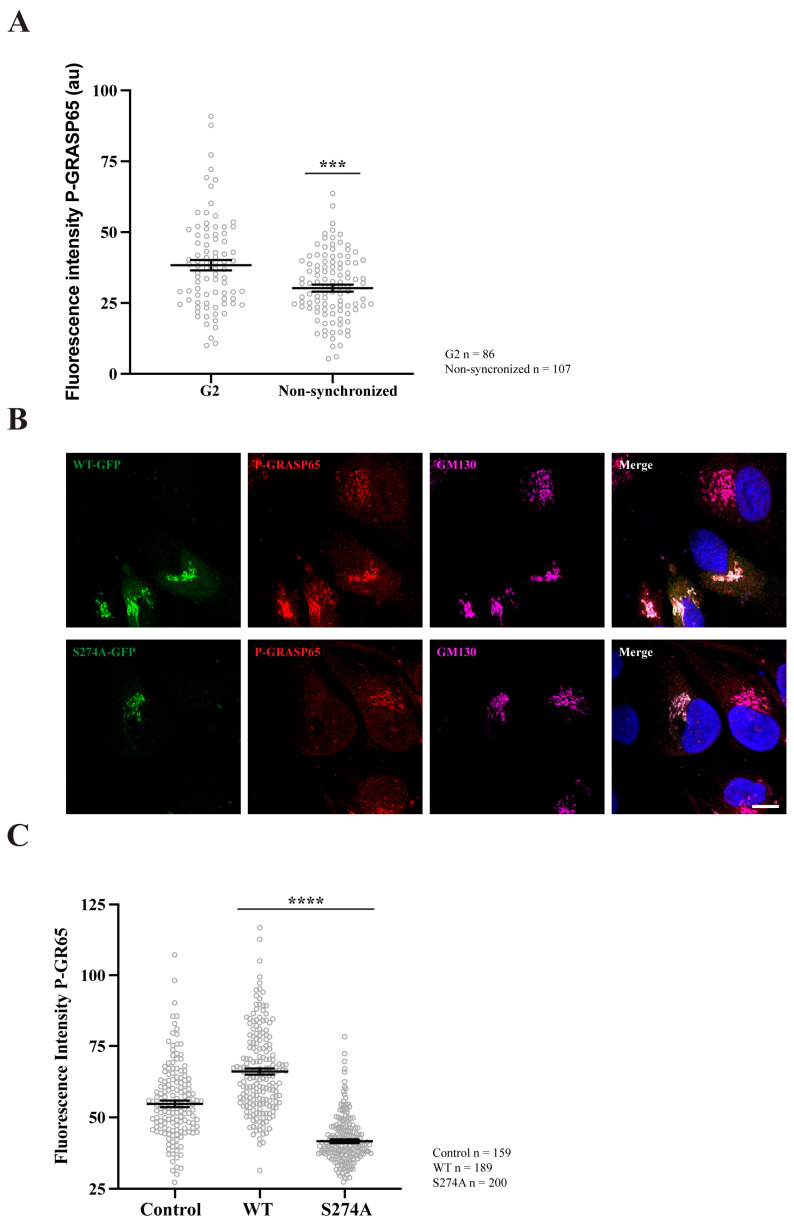
GRASP65 phosphorylation increases during G2 and after transfection with GRASP65-WT but is reduced in cells transfected with GRASP65-S274A. (**A**) HeLa cells, either synchronized in G2 using a double thymidine block or left unsynchronized, were fixed and stained with P-GRASP65 and GM130 antibodies. P-GRASP65 fluorescence intensity was quantified and normalized to GM130. In non-synchronized cells, the fluorescence intensity was 20% lower than in cells synchronized in G2. (**B**) Unsynchronized cells were plated and, after 24 h, transfected with EGFP-GRASP65-WT (WT) or EGFP-GRASP65-S274A (S274A). Twenty-four hours post-transfection, cells were fixed and processed for immunofluorescence. Representative images of cells expressing WT and S274A are shown. (**C**) Quantification revealed a 35% increase in P-GRASP65 fluorescence intensity with WT and a 40% reduction with S274A compared to untreated cells. Representative experiments out of two independent replicates. Fluorescence intensity values are expressed as means ± SEM from 15 broad fields per condition (**A**) or 40 broad fields per condition (**C**). In the latter case (**C**), control cells were non-transfected cells from the same coverslips. Statistical significance was assessed using Welch’s *t*-test: (**A**) non-synchronized cells vs. synchronized, *p* < 0.0003 marked by ***; (**C**) WT or S274A vs. untransfected cells, *p* < 0.0001 marked by ****. Scale bar: 5 µm.

We first verified the specificity of the antibody against phospho-GRASP65-S274 (hereafter referred to as P-GRASP65). Unless otherwise specified, cells were synchronized using a double thymidine block. HeLa cells were routinely plated on fibronectin-coated coverslips. Following synchronization, cells were fixed at the mitotic peak and processed for immunofluorescence analysis. Samples were co-stained with antibodies against P-GRASP65 and GM130, the latter serving as a reference for GC localization. Hoechst 33342 was used to stain the DNA. As illustrated in Figure 1, P-GRASP65 showed clear labeling at the GC during different phases of cell division. Next, plated cells were either synchronized or left unsynchronized. After fixation and labeling, the fluorescence intensity of P-GRASP65 was measured and normalized to that of GM130. As shown in Figure 2A, the fluorescence intensity of P-GRASP65 in G2-enriched cells increased by approximately 20% compared to that in asynchronous cells. This relatively modest effect can be attributed to the imperfect synchronization of the cell population with thymidine, which dilutes the G2-specific signal due to the presence of cells that are not actually in G2 [42], as addressed below in the Discussion. Notably, the single-cell data showed a broader distribution of fluorescence signals in the G2-enriched group, clustering at higher intensity values, likely corresponding to the subset of cells in the G2 phase. While most GRASP65 is bound to GM130 at the cis-Golgi, a small cytoplasmic fraction also exists [43]. The phosphorylated form localizes to the Golgi, as phospho-GRASP65 is detected there even in non-synchronized cells with intact Golgi ribbon, without increased cytoplasmic signal.

Subsequently, non-synchronized HeLa cells were plated and transfected with either EGFP-GRASP65-WT or a non-phosphorylatable GRASP65 mutant in which S274 was replaced with alanine (EGFP-GRASP65-S274A). The cells were then fixed and processed for immunofluorescence using antibodies against P-GRASP65 and GM130 (Figure 2B). Remarkably, WT-GRASP65 transfection led to a significant increase of 35% in P-GRASP65 signal intensity compared to non-transfected cells. Conversely, expression of the non-phosphorylatable S274A mutant resulted in a 40% decrease in the fluorescence intensity of P-GRASP65 relative to untreated cells (Figure 2C). This reduction, even lower than basal levels, is likely due to the displacement of endogenous GRASP65. These results further reinforce the phospho-specificity of the antibody.

**Figure 3:**
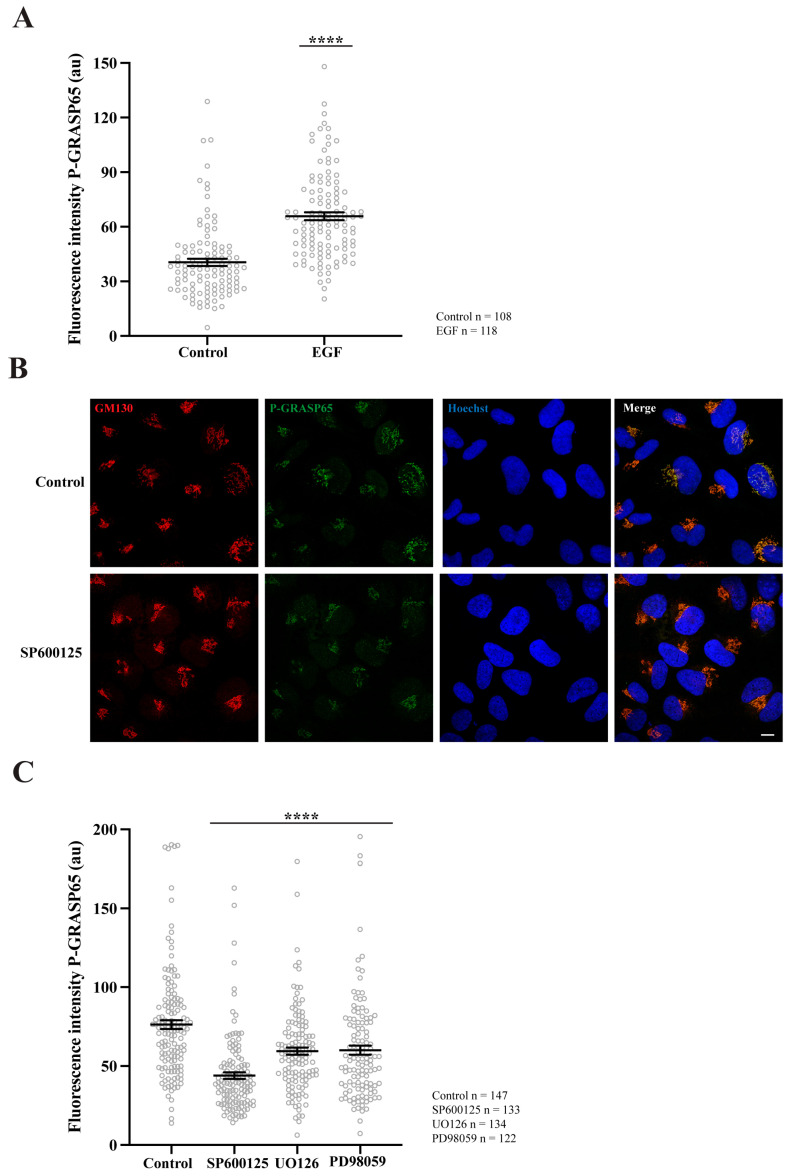
GRASP65 phosphorylation is enhanced by EGF, is reduced by JNK2 inhibition, and is minimally affected by MEK. (**A**) Non-synchronized cells were plated in complete medium and, the next day, switched to medium containing 1% FBS. After 24 h, they were incubated for an additional 24 h in serum-free medium. Cells were then treated with 50 ng/mL EGF for 10 min before fixation. P-GRASP65 fluorescence intensity increased by 40% following EGF treatment. (**B**) HeLa cells were synchronized using a double thymidine block. The JNK inhibitor SP600125 was added 4 h prior to fixation. Representative images of cells stained with antibodies against P-GRASP65 and GM130 and counterstained with Hoechst 33342. Inhibition of JNK by SP600125 results in a reduction of the P-GRASP65 signal. (**C**) Synchronized cells were treated with 50 µM SP600125 for 4 h or with 20 µM UO126 or 75 µM PD98059 for 2 h prior to fixation. Quantification of P-GRASP65 fluorescence intensity normalized to GM130 showed that JNK2 inhibition resulted in a 40% reduction in P-GRASP65 intensity compared to vehicle-treated control, while MEK inhibition caused a 20% decrease. Representative experiments out of three independent replicates. Fluorescence intensity values are expressed as means ± SEM from 15 broad fields per condition. Statistical significance was assessed using Welch’s *t*-test: (**A**,**C**) Untreated vs. EGF-treated cells and vehicle-treated cells vs. cells treated with SP600125, UO126 or PD98059, *p* < 0.0001 marked by ****. Scale bar: 10 µm.

To test if the P-GRASP65 signal was modulated, unsynchronized HeLa cells were maintained under low serum conditions and thereafter stimulated with EGF. This treatment resulted in a 40% increase in P-GRASP65 staining intensity (Figure 3A). This finding is consistent with that of Nakamura’s group, who developed a similar antibody to detect phosphorylation of the corresponding serine residue in the rat sequence of GRASP65 upon EGF or serum stimulation during interphase. However, in their study, this effect was mediated by ERK [44], while in G2, the phosphorylation is mediated by JNK2 [38]. As a proof-of-concept, we next assessed whether the P-GRASP65 signal was mediated by JNK in synchronized cells treated with the JNK inhibitor SP600125 (Figure 3B). Following fixation and staining, we quantified the fluorescence intensity of P-GRASP65. After normalization, we observed that SP600125 reduced P-GRASP65 levels by 40% compared with vehicle-treated control (Figure 3C), supporting the specificity of the signal and the involvement of residue S274 in JNK2-mediated phosphorylation. On the other hand, treatment with the MEK inhibitors UO126 and PD98059 resulted in a modest yet significant 20% inhibition compared to the control, suggesting that the MEK/ERK pathway plays a minor role in this process (Figure 3C). These findings establish the phospho-specific antibody as a robust tool for detecting GRASP65 phosphorylation in HeLa cells, providing the basis for elucidating the upstream regulatory network governing the JNK2-GRASP65 signaling axis that controls Golgi unlinking during G2.

### 3.2. PKD2 Inhibition or Depletion Reduces Phospho-GRASP65 Levels and Impairs Entry into Mitosis

Having established a reliable tool to detect GRASP65 phosphorylation in HeLa cells, we investigated its upstream regulation. Previous studies identified PKD as an upstream activator of the RAF1-MEK1 module [33], which governs the GC unlinking through ERK-mediated phosphorylation of GRASP55 [37,45]. Distinct from the role of ERK, our data indicated that GRASP65 is phosphorylated by JNK2 during the G2/M phase [38]. To examine whether the two GRASP orthologs are regulated by the same or distinct regulatory signaling pathways, we investigated whether PKD could also function as an upstream modulator of JNK2-mediated GRASP65 phosphorylation.

**Figure 4:**
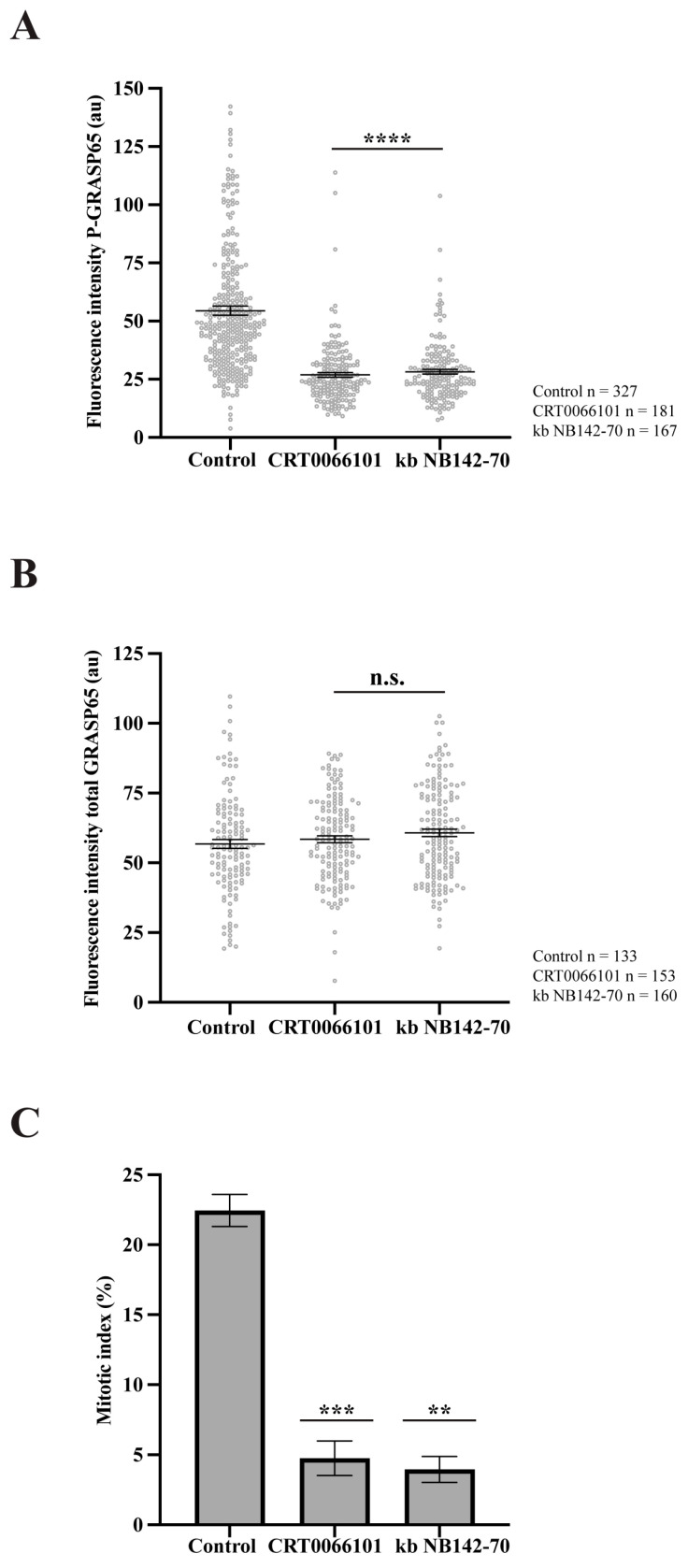
PKD inhibition reduces GRASP65 phosphorylation and mitotic entry. (**A**) HeLa cells were synchronized using a double thymidine block and treated with 5 µM CRT0066101 or 10 µM kb NB142-70 for 1 h prior to fixation. Cells were processed for immunofluorescence with antibodies against P-GRASP65 and GM130. Both PKD inhibitors reduced P-GRASP65 fluorescence intensity by 50%. (**B**) Cells were treated as in (**A**), then labeled with anti-GRASP65 and GM130 antibodies. GRASP65 fluorescence intensity was quantified and normalized to GM130. We did not observe differences in total GRASP65 levels. (**C**) HeLa cells were synchronized with the double thymidine block and fixed at the mitotic peak, 9 h after thymidine release. Inhibitors were added 1 h before fixation. Cells were labeled with antibodies against phospho-histone H3 and counterstained with Hoechst 33342 to quantify the mitotic index. Both inhibitors caused an 80% reduction in the mitotic index compared to the vehicle-treated control. Representative experiments out of three independent replicates. Fluorescence intensity (**A**,**B**) and percentage of mitotic cells (**C**) are expressed as means ± SEM from 15 broad fields per sample. Statistical significance was assessed using Welch’s *t*-test: (**A**) PKD2 inhibitors vs. control, *p* < 0.0001 marked by ****; (**B**) CRT0066101 or kb NB-142-70 vs. control were not statistically significant (n.s.); (**C**) CRT0066101 vs. control, *p* < 0.0005 marked by ***; and kb NB142-70 compared to the control, *p* < 0.0012 marked by **.

PKD2 and PKD3 are the predominant PKD isoforms expressed in HeLa cells [46,47]. Upon stimulation, PKD3 undergoes PKC-dependent translocation to the plasma membrane, followed by enhanced accumulation in the nucleus [48]. Accordingly, our study focused on PKD2, as it can localize at the GC [34]. To examine the role of this kinase, we synchronized HeLa cells and treated them with two structurally unrelated PKD inhibitors: CRT0066101 and kb NB142-70 [49,50]. Immunofluorescence analysis using antibodies against GM130 to label the GC and anti-P-GRASP65 revealed that both inhibitors caused a 50% reduction in P-GRASP65 fluorescence intensity compared with the control (Figure 4A). As a control, the treatment with CRT0066101 did not modify PKD levels (Supplementary Figure S2C). To rule out the possibility that the attenuation of the P-GRASP65 signal was due to a decrease in the total GRASP65, we measured its fluorescence intensity normalized with GM130. The results showed that the global GRASP65 levels were unaffected by these treatments (Figure 4B), indicating that the reduced P-GRASP65 signal reflected a specific decrease in GRASP65-S274 phosphorylation. In addition, we evaluated the effect of the inhibition of P-GRASP65 through both CRT0066101 and kb NB142-70 on the G2/M transition by quantifying the mitotic index. We observed an 80% decrease in the mitotic index with the two inhibitors (Figure 4C), in agreement with previous data [33,35].

**Figure 5:**
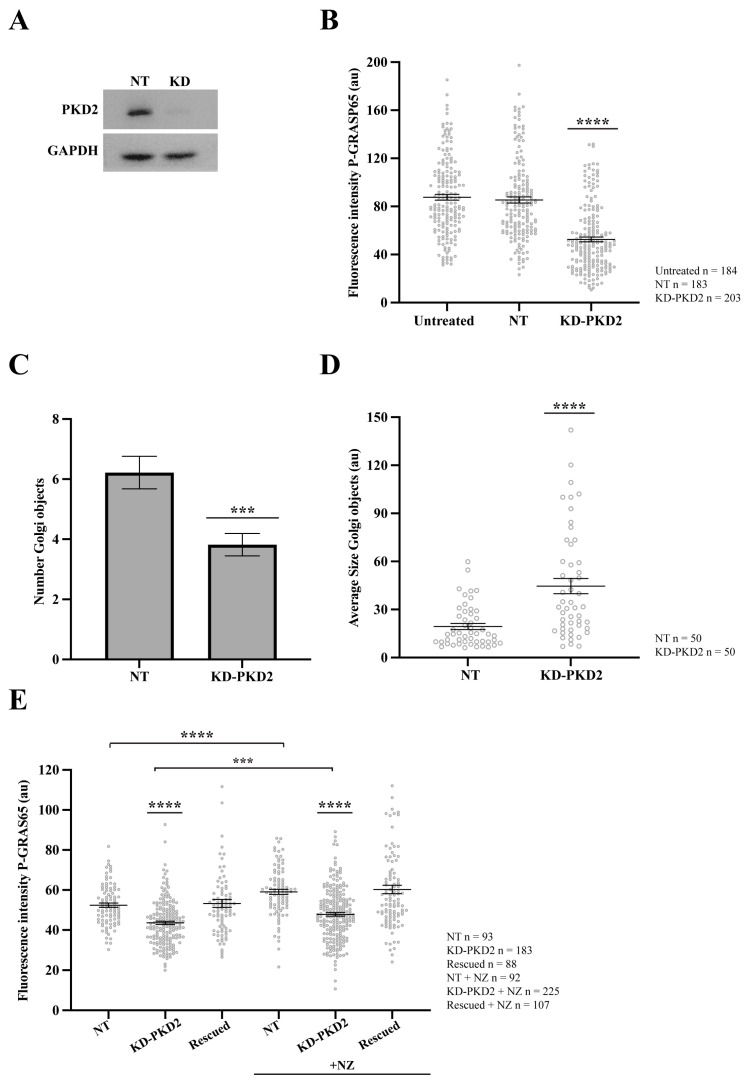
PKD2 knockdown reduces GRASP65 phosphorylation and Golgi unlinking. HeLa cells were transfected with siRNA targeting human PKD2 (KD-PKD2) or Non-Targeting (NT) control for 48 h. (**A**) Cells were lysed, and protein extracts were analyzed by SDS-PAGE followed by Western blotting. (**B**) NT-transfected and PKD2-silenced cells were synchronized using the double thymidine block. After fixation, cells were stained with antibodies against P-GRASP65 and GM130. Quantification of P-GRAS65 fluorescence intensity revealed a 40% reduction in KD-PKD2 compared to the NT control. (**C**) Quantification of the number of Golgi objects in synchronized cells showed a 35% reduction in PKD2-depleted cells compared to controls. (**D**) Measurement of the average size of Golgi objects indicated that Golgi fragments were twice as large in KD-PKD2 cells relative to the control. (**E**) Non-synchronized HeLa cells were first transfected with PKD2 siRNA, followed by transfection with a non-silencing PKD2 vector the next day (rescued). After 24 h, a subset of cells was treated with 5 µg/mL nocodazole for 30 min before fixation. Nocodazole (NZ)-treated control and rescued cells exhibited a 15% increase in P-GRASP65 fluorescence intensity compared to their respective untreated counterparts. In contrast, PKD2 knockdown cells, whether treated with NZ or not, showed a 20% and 15% reduction, respectively, relative to their corresponding controls. Representative data from three independent experiments. Fluorescence intensity quantification was based on 15 broad fields per sample (**B**) or 40 broad fields (**E**). In the latter case (**E**), PKD2 knockdown cells were untransfected cells from the same coverslip that had been depleted and subsequently transfected with the non-silencing vector. Quantification of the number of Golgi objects and average size (**C**,**D**) was based on the analysis of 50 cells per sample. Data are expressed as means ± SEM. Statistical significance was assessed using Welch’s *t*-test: (**B**) NT vs. KD-PKD2, *p* < 0.0001 marked by ****; (**C**) NT vs. KD-PKD2, *p* < 0.0004 marked by ***; (**D**) NT vs. KD-PKD2, *p* < 0.0001 marked by ****; (**E**) comparisons of KD-PKD2 with or without NZ to their respective controls, and NT untreated vs. NZ-treated NT, *p* < 0.0001, marked by ****; and KD-PKD2 with NZ vs. untreated KD-PKD2 cells, *p* < 0.0005 marked by ***.

To confirm the specificity of the pharmacological inhibition, we evaluated the effects of the siRNA-mediated PKD2 depletion on the P-GRASP65 levels and GC organization in synchronized HeLa cells. As shown in Figure 5A, siRNA-mediated knockdown of PKD2 led to a more than 90% reduction in the protein levels, as determined by Western blotting. This was accompanied by a 40% decrease in the GM130-normalized P-GRASP65 fluorescence intensity compared to non-targeting (NT) siRNA-transfected cells (Figure 5B). Similar results were obtained using an unrelated siRNA sequence to knock down PKD2, although this was less efficient, as it reduced the P-GRASP65 fluorescence intensity by 25% (Supplementary Figure S1A,B), thus confirming the effects observed with both PKD inhibitors. Notably, following PKD2 depletion, we measured an approximately 35% reduction in the number of Golgi objects compared to the NT-transfected cells (Figure 5C and Supplementary Figure S1C) and a twofold increase in their average size with respect to the control (Figure 5D). Collectively, these results indicate that siRNA-mediated depletion of PKD2 reduced Golgi unlinking during the G2 phase of the cell cycle via GRASP65.

Additionally, the role of PKD2 was investigated in asynchronous cells subjected to siRNA-mediated knockdown of PKD2. Following depletion, the cells were transfected with an siRNA-resistant PKD2 construct (non-silencing PKD2) to assess a functional rescue. Before fixation, a subset of cells was treated for 30 min with the microtubule-depolymerizing agent nocodazole, a well-established inducer of Golgi unlinking and PKD activation [51]. Quantitative analysis of P-GRASP65 fluorescence intensity normalized with GM130 showed that the treatment of the NT control with nocodazole increased the phosphorylation signal by 15% compared to the untreated control. In contrast, PKD2 knockdown, whether treated with nocodazole or left untreated, reduced the levels of P-GRASP65 compared to their corresponding NT controls. Specifically, this reduction was 20% and 15%, respectively. Importantly, the transfection with the siRNA-resistant PKD2 following the siRNA-mediated knockdown restored GRASP65 phosphorylation at S274 under both conditions to levels similar to the respective controls, supporting a direct functional link between PKD2 activity and GRASP65 phosphorylation (Figure 5E).

Taken together, these results demonstrate that PKD2 is required for GRASP65 phosphorylation at S274 and Golgi unlinking during the G2 phase. Moreover, PKD2 depletion inhibited GRASP65 phosphorylation both in synchronized cells (Figure 5B) and in non-synchronized cells exposed to nocodazole (Figure 5E), suggesting that PKD2 plays a broader role in regulating GRASP65 phosphorylation beyond cell cycle-associated functions. These findings indicate that PKD2 is a central upstream regulator of the JNK2-GRASP65 module, providing a framework for dissecting the organization of signaling pathways controlling Golgi dynamics. The reduction in the unlinking observed after depletion of PKD2 is in agreement with previous data that demonstrate that siRNA-mediated knockdown of PKD induces a delay in the G2/M transition [33,35,36]. Strikingly, given that PKD has previously been shown to regulate the RAF1-MEK1-ERK axis [33], which in turn induces GRASP55 phosphorylation and Golgi unlinking [37,45], these findings raise the compelling possibility that PKD2 functions as a shared upstream hub coordinating the phosphorylation of both GRASP paralogs, GRASP55 and GRASP65, through distinct but parallel downstream effector cascades, namely ERK and JNK2 respectively.

### 3.3. PKD2 Inhibition Reduces GRASP65 Phosphorylation in Pharmacologically G2-Arrested Cells

**Figure 6:**
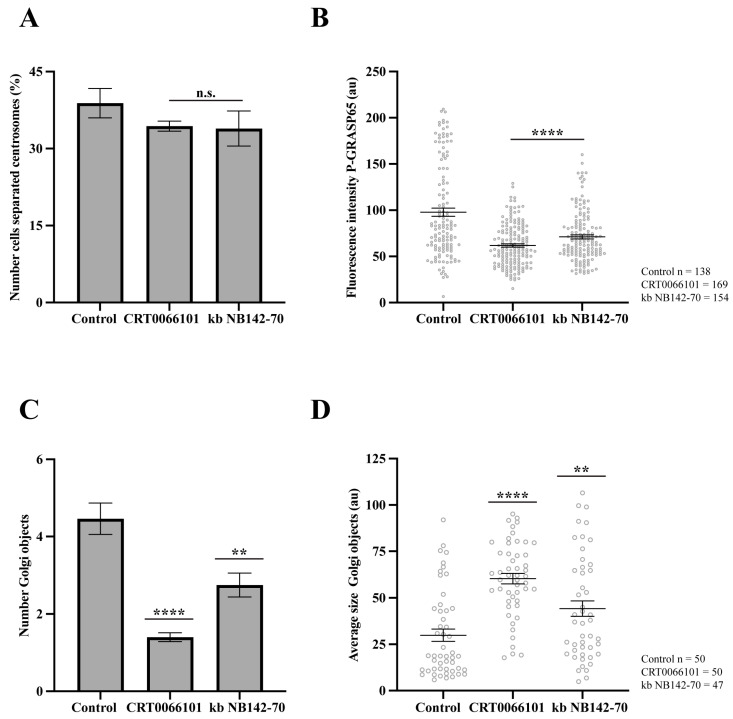
Effect of inhibitors of PKD2 in pharmacologically G2-arrested cells. HeLa cells were treated with 9 µM RO-3306 for 20 h to induce G2 arrest. (**A**) HeLa cells were treated with 5 µM CRT0066101 and 10 µM kb NB142-70 for 1 h before fixation, then were fixed and labeled with anti-pericentrin, anti-GM130 and Hoechst 33342. The percentage of cells with separated centrosomes was quantified using maximum-intensity projections of z-stacks. No significant differences were observed between treated cells and controls. (**B**) Cells treated as in (**A**) were stained with antibodies against P-GRASP65 and GM130. Both CRT0066101 and kb NB142-70 reduced P-GRASP65 fluorescence intensity by 40 and 30%, respectively. (**C**) Quantification of the number of Golgi objects showed that CRT0066101 caused a 70% reduction, whereas kb NB142-70 induced a 35% reduction compared to the control. (**D**) The average size of Golgi objects increased significantly: CRT0066101 caused a twofold increase, while kb NB142-70 induced a 40% increase compared to control. Representative experiment out of three independent replicates. The percentage of cells with separated centrosomes and the fluorescence intensity of P-GRASP65 were calculated from 15 broad fields (**A**,**B**). For quantification of Golgi objects and average size, 50 cells per sample were analyzed (**C**,**D**). Data are expressed as means ± SEM. Statistical significance was assessed using Welch’s *t*-test: (**A**) cells treated vs. control, n.s.; (**B**) inhibitors vs. control, *p* < 0.0001 marked by ****; (**C**) control vs. CRT0066101, *p* < 0.0001 marked by **** and control vs. kb NB142-70, *p* < 0.0012 marked by **; (**D**) control vs. CRT0066101, *p* < 0.0001 marked by **** and control vs. kb NB142-70, *p* < 0.0085 marked by **.

Once it was established that PKD inhibition reduces GRASP65 phosphorylation and impairs the G2/M transition, we considered the possibility that the observed decrease in P-GRASP65 levels might be an indirect effect of reduced entry into G2. Thus, we first examined whether these inhibitors could interfere with cell accumulation in the G2 phase. To specifically address this, HeLa cells were arrested in G2 by a 20-hour treatment with the CDK1 inhibitor RO-3306 [41]. One hour before fixation, the cells were exposed to vehicle (control) or the PKD inhibitors CRT0066101 and kb NB142-70. For this purpose, following fixation, we labeled the cells with anti-pericentrin antibodies, GM130 and Hoechst. Subsequently, maximum-intensity projections were generated from z-stacks of fluorescence microscopy images. The percentage of cells exhibiting separated centrosomes was then quantified, because centrosome separation occurs only after entry into the G2 phase [52]. As shown in Figure 6A, neither inhibitor affected centrosome separation, indicating that the transition from S to G2 was not impaired. Then, immunofluorescence examination was performed using antibodies against P-GRASP65 and GM130. Quantitative analysis of P-GRASP65 fluorescence intensity revealed that both inhibitors induced a consistent reduction in the signal, ranging from 30% to 40% compared with the vehicle-treated control, even in previously G2-arrested cells (Figure 6B). In addition, after treatment with CRT0066101 and kb NB142-70, we observed a 70% and a 35% decrease in the number of Golgi objects, respectively (Figure 6C), accompanied by a significant increase in their average size, in particular a twofold increase with CRT0066101 and a 40% increase with kb NB142-70 (Figure 6D). Collectively, these findings indicate that PKD2 inhibition reduces the extent of Golgi ribbon unlinking in G2-arrested cells. Altogether, these findings indicate that the inhibition of PKD does not perturb G2 accumulation of the cells and that the observed reduction in P-GRASP65 levels reflects a direct regulatory role of PKD2, rather than a secondary consequence of an altered S-to-G2 cell cycle progression.

Another important question is whether inhibition of GRASP65 phosphorylation could be a side effect of signaling events downstream of the Golgi unlinking, since Golgi disassembly into isolated stacks during G2 has been shown to activate Src [53], which can modulate PKD. Specifically, phosphorylation of key residues on PKD can coordinate different cellular functions, including direct activation of the kinase [54], response to oxidative stress [55], and cell adhesion [54]. Thus, we tested whether Src activity was required for GRASP65 phosphorylation. To this end, the cells were synchronized in G2 by a double thymidine block and subsequently treated with the structurally unrelated Src inhibitors PP2 and Dasatinib for 2 and 24 h, respectively, prior to fixation. Immunofluorescence analysis was performed using anti-phospho-GRASP65 antibodies and GM130 staining to visualize the GC. As shown in Supplementary Figure S2A,B, the quantitative analysis showed that Src inhibition did not affect GRASP65 phosphorylation. These findings suggest that the reduction in GRASP65 phosphorylation upon PKD inhibition is not an indirect effect of impaired Golgi unlinking. Collectively, our results establish a direct link between PKD activation and GRASP65 phosphorylation during G2.

### 3.4. PKD2 Activation Increases GRASP65 Phosphorylation

**Figure 7:**
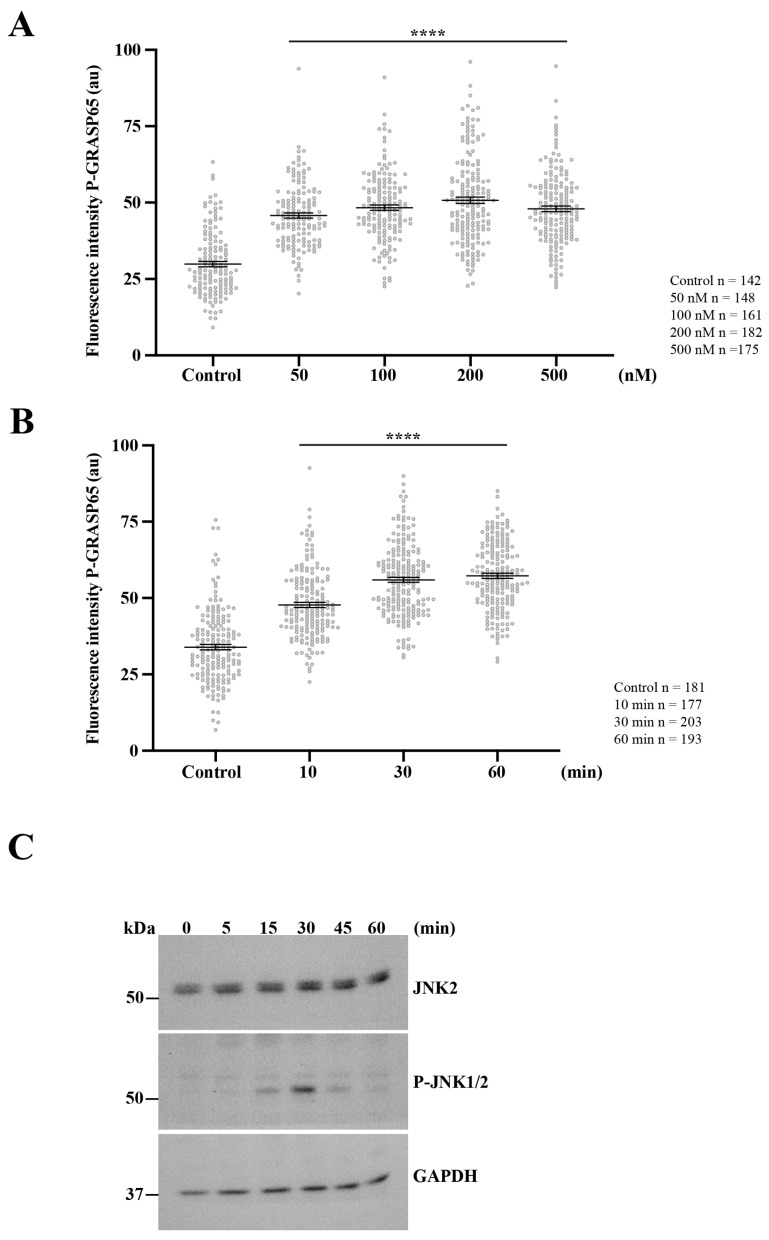
Activation of PKD2 enhances JNK2-mediated GRASP65 phosphorylation. (**A**) Unsynchronized HeLa cells were exposed to increasing concentrations of PMA for 20 min, then were processed for immunofluorescence. P-GRASP65 fluorescence intensity was quantified and normalized to GM130. GRASP65 phosphorylation increased with PMA concentration and plateaued between 100 and 200 nM. (**B**) We performed a time-course experiment using 200 nM PMA for the indicated time points. Cells were processed for immunofluorescence and quantified as in (**A**). P-GRASP65 fluorescence intensity peaked at 30 min following PMA treatment. (**C**) Cells were treated with 200 nM PMA, lysed at the indicated time points, and analyzed by SDS-PAGE and Western blotting. Maximal phosphorylation of JNK1/2 was observed at 30 min. Representative results from two independent experiments. Quantification of P-GRASP65 fluorescence intensity was based on 15 broad fields per sample. Data are expressed as means ± SEM. Statistical significance was assessed using Welch’s *t*-test: (**A**,**B**) *p* < 0.0001 marked by ****.

To further substantiate the role of PKD2 in modulating JNK2-dependent GRASP65 phosphorylation, we examined whether the pharmacological activation of PKD influences the phosphorylation status of both JNK2 and GRASP65. To this end, unsynchronized HeLa cells were treated with increasing concentrations of phorbol 12-myristate 13-acetate (PMA), a well-established activator of the PKC/PKD pathway [56], for 20 min. The cells were then fixed and processed for immunofluorescence using antibodies against phospho-GRASP65 and GM130 to evaluate the normalized phosphorylation levels of GRASP65 within the GC. This treatment induced a robust increase in P-GRASP65 signal intensity, which plateaued between 100 and 200 nM (Figure 7A).

Next, we investigated the kinetics of this response by treating non-synchronized cells with PMA for different time intervals. The quantification showed that the P-GRASP65 levels reached a maximum after 30 min of treatment (Figure 7B). This increase closely paralleled JNK activation, as detected by Western blotting using phospho-JNK antibodies in lysates of unsynchronized cells treated with 200 nM of PMA over a time course (Figure 7C).

Collectively, these data demonstrate that PKD activation is sufficient to promote GRASP65 phosphorylation at S274 via the JNK pathway, supporting a model in which PKD2 functions as a regulator of the signaling cascade that drives Golgi ribbon unlinking during cell cycle progression. These findings establish a direct mechanistic link between PKD2 and the JNK2-GRASP65 axis, highlighting the existence of a PKD-dependent signaling hub that coordinates cell cycle cues with the structural remodeling of the Golgi apparatus. Moreover, the responsiveness of this pathway to phorbol esters indicates that PKD2 leads to JNK2 activation to induce GRASP65 phosphorylation.

## 4. Discussion

Our findings show that PKD controls, through JNK2, the phosphorylation of serine 274 of GRASP65, a modification that plays a crucial role in promoting the separation of the Golgi ribbon into isolated stacks, a process that is required for G2/M transition and spindle formation [20]. Remodeling of the GC structure also occurs in response to a wide range of physiological and pathological conditions, including differentiation [57], migration [58], oxidative stress [59,60], aging [61], viral infection [62,63], and cancer [64]. Therefore, understanding the molecular mechanisms that regulate GC organization may help identify novel therapeutic targets for disease processes characterized by GC fragmentation.

GRASP65 regulates GC organization through its role in tethering Golgi membranes and by promoting the stabilization and, consequently, the acetylation of Golgi-associated microtubules [40]. Both acetylation of microtubules and tethering are inhibited by JNK2-mediated phosphorylation of serine 274, leading to Golgi ribbon unlinking [38]. Given the central role of this modification in controlling Golgi architecture, understanding how GRASP65 phosphorylation is regulated is key to elucidating the signaling pathways that govern Golgi dynamics. To this end, we developed and validated a phospho-specific antibody targeting serine 274 of GRASP65, which enabled precise detection of its phosphorylation status by immunofluorescence microscopy. The antibody proved to be highly specific and sensitive to changes in signaling activity, allowing assessment of GRASP65 phosphorylation under various experimental conditions. The P-GRASP65 signal was specific, as it decreased when cells were transfected with the non-phosphorylatable EGFP-GRASP65-S274A mutant, whereas it increased markedly following transfection with the WT construct. The anti-phospho-GRASP65 signal was strongly reduced by a JNK inhibitor and, to a lesser extent, by two MEK-blocking compounds. In contrast, its levels increased during G2. However, the signal was also detectable in non-synchronized cells, consistent with our previous finding that the steady-state structure of the Golgi ribbon results from an equilibrium between the formation and cleavage of membrane tubules in the non-compact zones [65], suggesting that GRASP65 phosphorylation and dephosphorylation likely occur continuously. The modest increase in P-GRASP65 fluorescence intensity observed in double thymidine-synchronized cells, compared to unsynchronized cells, may reflect the influence of additional regulatory mechanisms acting on GRASP65 phosphorylation. In non-synchronized cultures, serum growth factors present in the complete medium can activate signaling pathways, such as ERK [44], which promote GRASP65 phosphorylation independently of the cell cycle phase. In addition, within the asynchronous population, a subset of cells may already have entered G2/M, contributing to higher baseline levels of phosphorylated GRASP65. Moreover, synchronization after thymidine release is often imperfect, as cells progress through the cycle with a degree of temporal variation, resulting in partial overlap between phases [42]. Collectively, these regulatory and temporal influences likely underlie the modest difference in fluorescence intensity observed between synchronized and asynchronous cells.

The use of our phospho-specific antibody allowed us to investigate the upstream regulation of GRASP65 phosphorylation. Using this approach, we found that pharmacological inhibition of PKD with two structurally unrelated compounds caused a marked reduction in P-GRASP65 levels, which correlated with decreased Golgi unlinking and impaired G2/M transition, in agreement with previous observations [33,35]. Similarly, siRNA-mediated knockdown of PKD2 recapitulated these phenotypes, which were rescued by the expression of an siRNA-resistant form of PKD2, confirming the specificity of the observed effects. Therefore, these results identify PKD as a critical upstream regulator of GRASP65 phosphorylation.

In addition, we performed control experiments to exclude indirect causes for the reduction in P-GRASP65 upon PKD inhibition. Specifically, we found that total GRASP65 levels remained unchanged, indicating that the decreased phospho-signal was not due to protein degradation. As PKD2 depletion causes a delay in mitotic entry, we arrested cells at the G2/M transition using the CDK1 inhibitor RO-3306 and observed that P-GRASP65 levels were still reduced following PKD inhibition, demonstrating that this effect was not a secondary consequence of G2/M arrest or of reduced centrosome separation. Indeed, PKD inhibition did not interfere with G2 accumulation, as shown by quantification of cells with separated centrosomes, a hallmark of late G2 [52].

Moreover, inhibition of Src signaling, which is activated upon Golgi fragmentation during G2 [20], did not affect P-GRASP65 levels. These results suggest that PKD2 regulates GRASP65 independently of signaling events triggered by structural changes in the GC.

Finally, we showed that stimulation of PKD activity with PMA caused a rapid and robust increase in both P-GRASP65 and phospho-JNK levels, consistent with findings that JNK, in addition to ERK, acts as a signaling mediator of PKC and is phosphorylated in response to PMA [66]. Altogether, our data indicate a direct signaling axis involving PKD2, JNK2 and GRASP65 that promotes the unlinking of the Golgi ribbon during the G2 phase, a prerequisite for proper mitotic progression. As the sequence surrounding serine 274 does not match the consensus sequence for the PKCμ/PKD family, LxRxxS/T [67], a direct phosphorylation of this site by PKD is unlikely. However, although it cannot be excluded that PKD phosphorylates additional sites on GRASP65, our model suggests that PKD induces JNK2 activation through upstream MAP kinase cascade components and that active JNK2 then phosphorylates GRASP65 at serine 274.

Under basal conditions, PKD1 and PKD2 are primarily localized in the cytoplasm. However, upon stimulation with ligands such as G protein-coupled receptor (GPCR) agonists, they rapidly and reversibly translocate to the plasma membrane, where they participate in downstream signaling cascades [68,69]. After activation, PKD isoforms can also localize to subcellular compartments, most notably the *trans*-Golgi network (TGN), where they regulate the fission of post-Golgi cargo carriers. Additionally, PKD2 localizes to centrosomes, mitotic spindles, and midbodies [36]. At the centrosomes, PKD stabilizes Aurora A, protecting it from ubiquitination and proteasomal degradation [35]. By contrast, PKD3, in resting cells, exhibits both cytoplasmic and nuclear localization due to active nucleocytoplasmic shuttling [48]. Central to the translocation of PKD to membranes is diacylglycerol (DAG), a lipid second messenger that binds to the C1a cysteine-rich domain of PKD [70], thereby recruiting the kinase to membranes and initiating its conformational activation. DAG is generated locally, for instance at the GC by PLCβ3, which is activated downstream of heterotrimeric G protein βγ subunits (particularly β1γ2 and β3γ2) released upon GPCR stimulation. DAG also recruits PKCη, which phosphorylates PKD1 at serines 744 and 748, leading to its full activation. This Gβγ-PLCβ3-DAG-PKCη-PKD pathway is essential for Golgi membrane fission and the formation of transport carriers [71,72,73]. Crosstalk with other regulators, such as phosphatidylinositol 4-phosphate (PI4P) and its associated enzymes (PI4KIIIβ and CERT), further links PKD2 to lipid-mediated control of GC organization [74,75]. Moreover, several small GTPases contribute to the spatial and functional regulation of PKD at the GC. For example, ARF1 binds directly to proline 275 within the second C1b cysteine-rich domain of PKD2, facilitating its recruitment to the TGN [76]. RhoA promotes PKD activation via PKC, thereby linking actin dynamics to Golgi remodeling [77,78], while Rab6, although not a direct activator, localizes to post-Golgi compartments and may influence PKD-dependent trafficking [79,80]. Collectively, these and other regulatory inputs ensure the precise activation of PKD in response to diverse signaling pathways.

A critical question concerns the mechanism by which PKD2 is activated during the G2 phase. One possibility is that partial microtubule depolymerization during G2 triggers the release of GEF-H1, leading to RhoA activation [81,82,83]; this, in turn, could promote PLCε activation and local DAG production, thereby recruiting and activating PKD [78].

Alternatively, disassembly of focal adhesions, which is regulated by DEPDC1B through competition with RhoA for binding to Protein Tyrosine Phosphatase Receptor Type F (PTPRF) [84], may influence Golgi remodeling. RhoA is known to activate PKD through multiple signaling pathways, with the exact mechanism being cell-type dependent [80,85,86]. Although not directly examined in this study, these upstream cues may converge on PKD2 during G2 to initiate GC remodeling. Our phospho-specific GRASP65 antibody now provides a valuable tool to further dissect these upstream regulatory mechanisms in detail.

Another question is how PKD activates JNK2. While PKD can activate the RAF1–MEK1-ERK signaling cascade [33], which likely targets GRASP55 [31,45], we hypothesize that PKD2 could function as a regulator of one or more MAP3Ks. These, in turn, may activate JNK2 through MKK4 and MKK7, thereby initiating G2-specific signaling events. Significantly, several MAP3Ks are localized to intracellular membranes, including the GC. For instance, MAP3K11 (MLK3) plays a role in the G2/M transition and can activate JNK through MKK4 and/or MKK7. Indeed, MLK3 localizes with Golgin 160 on the *cis* side of the GC [87]. In this context, Cdc42 and Rac1 may also be implicated, as both can bind MLK3 to promote JNK activation [88]. Another MAP3K implicated in JNK activation is MAP3K12 (DLK), which is partially localized to the GC in different cell types [89,90,91]. However, so far, we cannot yet rule out the involvement of other MAP3Ks upstream of JNK, beyond those mentioned above, and not necessarily localized at the GC. Moreover, although our previous results showed that JNK2 can localize to the GC [38], suggesting that PKD2 might also act at this site, it cannot be excluded that PKD2 does not necessarily reside at the GC and may instead activate signaling pathways that lead to JNK2 activation from other cellular locations.

Additionally, the death-associated protein kinase (DAPK) modulates PKD signaling and is required for JNK activation in response to oxidative stress [92]. DAPK is also implicated in cell cycle regulation; it localizes to centrosomes and colocalizes and interacts with PLK1 during mitotic progression [93]. Similarly, phosphorylation of the serine189 in GRASP65 by PLK1 is necessary for Golgi ribbon unlinking in late G2 [94]. In addition, we cannot exclude the involvement of p38 MAPK, as reciprocal regulation between PKD and p38 has been reported in a context-dependent manner [95,96]. Future studies will aim to elucidate the role of these kinases in the PKD-regulated, GRASP65-dependent unlinking of the GC during G2.

## 5. Conclusions

In conclusion, our findings identify PKD2 as a key integrator of Golgi-based signaling during G2, directing the phosphorylation of GRASP65 at serine 274, a critical event required for Golgi unlinking and cell cycle progression. A major advance of this study is the development of a phospho-specific antibody against GRASP65, which provides a powerful tool to monitor the activation state of this signaling pathway with high spatial and temporal resolution. This antibody enables detailed dissection of upstream regulatory mechanisms and serves as a potential biomarker for Golgi remodeling events in both physiological and pathological contexts. Given that dysregulated Golgi dynamics are increasingly recognized as a hallmark of various diseases, our work sets the foundation for future investigations into GRASP65-driven signaling networks that couple membrane remodeling to cell fate decisions.

## Data Availability

The datasets generated and/or analyzed during the current study are not publicly available due to the novelty of the identified upstream signaling pathway involved in GRASP65 phosphorylation during the G2 phase of the cell cycle but are available from the corresponding author upon reasonable request.

## Supplementary Materials

The following supporting information can be downloaded at https://www.mdpi.com/article/10.3390/cells15060565/s1, Figure S1: PKD2 knockdown using an alternative siRNA also reduced GRASP65 phosphorylation. (A) Cell extracts of HeLa cells were analyzed by SDS-PAGE and Western blotting. siRNA-mediated knockdown of PKD2 resulted in more than a 90% reduction of protein levels. (B) Synchronized cells were fixed and stained with antibodies against P-GRASP65 and GM130. P-GRASP65 fluorescence intensity was quantified and normalized to that of GM130. Although the effect was less pronounced, a 25% of reduction of P-GRASP65 fluorescence intensity was observed. Representative results from two independent experiments. Fluorescence intensity quantification was based on 15 broad fields per sample. Data are expressed as means ± SEM. Statistical significance was assessed using Welch’s *t*-test: (B) *p* < 0.0001 marked by ****. (C) For Golgi objects quantification, the three image channels (upper panels) were splitted and converted to 8-bit using Fiji/ImageJ. Images were thresholded and converted into binary masks, which were subsequently analyzed using the “Analyze Particles” function to identify Regions of Interest (ROI). Finally, 50 cells from 15 randomly selected independent fields were analyzed. Scale bar: 10 μm. Figure S2: GRASP65 phosphorylation is independent of Src. HeLa cells were synchronized using the double thymidine block. Prior to fixation, cells were treated with 10 μM PP2 for 2 h (A) or 5 μM Dasatinib for 24 h (B). Cells were processed for immunofluorescence, and the P-GRASP65 fluorescence intensity was quantified and normalized to GM130. Representative results from two independent experiments. Data are expressed as means ± SEM from 15 broad fields per sample. Statistical significance was assessed using Welch’s *t*-test: Control vs PP2 or Dasatinib were not statistically significant (n.s.). (C) Western blots showing the levels of PKD2 and GAPDH in HeLa cell lysates treated or not for 1 h with 5 μM CRT0066101.

## Abbreviations

The following abbreviations are used in this manuscript:

DAG	Diacylglycerol
EGF	Epidermal growth factor
GC	Golgi complex
GEF	Guanine nucleotide exchange factor
JNK2	c-Jun N-terminal kinase 2
MAPK	Mitogen-activated protein kinase
MEK	MAPK/ERK kinase
MTOC	Microtubule-organizing center
P-GRASP65	GRASP65 phosphorylated at serine 274
PH	Pleckstrin homology domain
PKC	Protein kinase C
PKD	Protein kinase D
PKD2	Protein kinase D isoform 2
PLC	Phospholipase C
PMA	Phorbol 12-myristate 13-acetate
SPR	Serine-proline-rich domain
