# Supplementary material for: Protein Kinase D2 Regulates GRASP65 Phosphorylation and Golgi Ribbon Unlinking During G2/M Transition
Source: Cells. 2026 Mar 21;15(6):565. doi: 10.3390/cells15060565 (PMC13025926; doi:10.3390/cells15060565)
Supplement: Supplementary file 1 [file cells-15-00565-s001.zip › cells-4180955-supplementary.pdf]

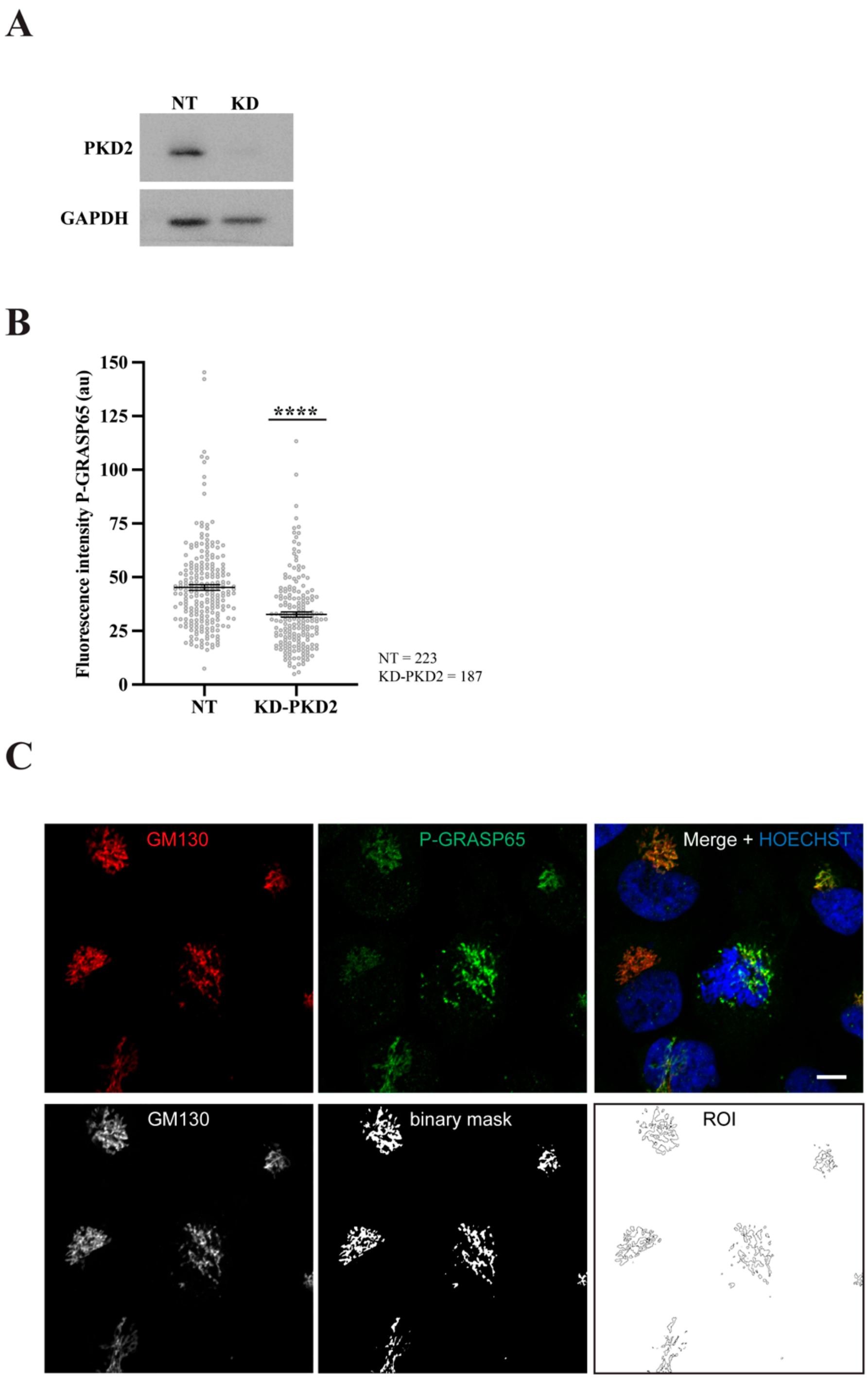

Supplementary Figure S1. PKD2 knockdown using an alternative siRNA also reduced GRASP65 phosphorylation. (A) Cell extracts of HeLa cells were analyzed by SDS-PAGE and Western blotting. siRNA-mediated knockdown of PKD2 resulted in more than a 90% reduction of protein levels. (B) Synchronized cells were fixed and stained with antibodies against P-GRASP65 and GM130. P-GRASP65 fluorescence intensity was quantified and normalized to that of GM130. Although the effect was less pronounced, a 25% of reduction of P-GRASP65 fluorescence intensity was observed. Representative results from two independent experiments. Fluorescence intensity quantification was based on 15 broad fields per sample. Data are expressed as means ± SEM. Statistical significance was assessed using Welch's t-test: (B) $P < 0.0001$ marked by ****. (C) For Golgi objects quantification, the three image channels (upper panels) were splitted and converted to 8-bit using Fiji/ImageJ. Images were thresholded and converted into binary masks, which were subsequently analyzed

using the “Analyze Particles” function to identify Regions of Interest (ROI). Finally, 50 cells from 15 randomly selected independent fields were analyzed. Scale bar: 10 μm.

A

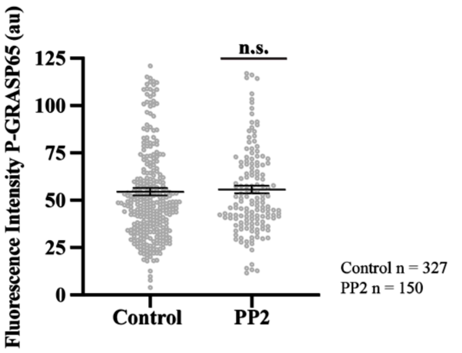

B

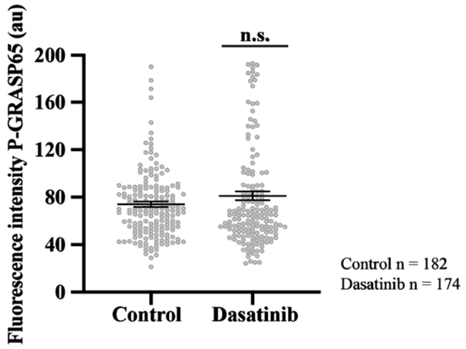

C

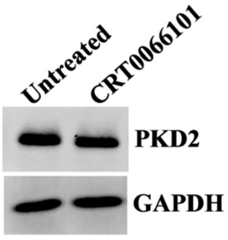

Supplementary Figure S2. GRASP65 phosphorylation is independent of Src. HeLa cells were synchronized using the double thymidine block. Prior to fixation, cells were treated with 10 μM PP2 for 2 h (A) or 5 μM Dasatinib for 24 h (B). Cells were processed for immunofluorescence, and the P-GRASP65 fluorescence intensity was quantified and normalized to GM130. Representative results from two independent experiments. Data are expressed as means ± SEM from 15 broad fields per sample. Statistical significance was assessed using Welch’s t-test: Control vs PP2 or Dasatinib were not statistically significant (n.s.). (C) Western blots showing the levels of PKD2 and GAPDH in HeLa cell lysates treated or not for 1 h with 5 μM CRT0066101.
